# Adrenocortical Secreting Mass in a Patient with Gardner's Syndrome: A Case Report

**DOI:** 10.1155/2010/682081

**Published:** 2010-12-27

**Authors:** Nabila Mejdoub Rekik, Sourour Ben Salah, Nozha Kallel, Mahdi Kamoun, Nadia Charfi, Mohamed Abid

**Affiliations:** Department of Diabetes and Endocrinology, Hedi Chaker Hospital, Magida Boulila Avenue. Sfax 3029, Tunisia

## Abstract

Gardner's syndrome (GS) is a dysplasia characterized by neoformations of the intestine, soft tissue, and osseous tissue. Endocrine neoplasms have occasionally been reported in association with GS. Adrenal masses in GS are rare, and few have displayed clinical manifestations. In the current paper, The authors report a 37-year-old male patient with GS including familial adenomatous polyposis (FAP) and mandible osteoma who presented with an incidental adrenal mass. Computerized tomography adrenal scan identified bilateral masses. Functional analyses showed a hormonal secretion pattern consistent with pre-Cushing's syndrome. Other extraintestinal manifestations were hypertrophy of the pigmented layer of the retina and histiocytofibroma in the right leg. This paper describes a rare association of adrenocortical secreting mass in an old male patient with Gardner syndrome.

## 1. Introduction

Gardner's syndrome (GS) is a variant of familial adenomatosis polyposis (FAP), which affects one in 8300 individuals and one in 7500 births in the United States [1]. The disease is characterized by colonic polyps and extracolonic manifestations. The polyps typically develop in adolescence and undergo malignant change between the third and fifth decades of life. The extracolonic manifestations include osteomas, desmoids tumors, epidermoid cysts, and malignancies [2].

It is believed that GS and familial adenomatous polyposis are variants of the same disorder, since they share the same genetic alterations [3]. The fact that GS is associated with extracolonic manifestations may be explained by a variable penetrance of a common mutation. The disorder is linked to band 5q21-q22, the adenomatous polyposis coli locus (APC gene) [4]. More than 1400 different mutations of this gene have been reported. These mutations have a nearly complete penetrance of the colonic phenotype, but a variable penetrance of the extracolonic manifestations of the disease [4].

Among rare extracolonic manifestations of AFP, endocrine neoplasms of the adrenal cortex have occasionally been reported.

We describe a pre-Cushing's syndrome in a 37-year-old male patient with GS who presented with an incidental adrenal mass.

## 2. Case Report

A 37-year-old male with a 6-year history of AFP syndrome was referred to our endocrinologist outpatients for evaluation of an adrenal mass.

In 2003, the patient was found to have multiple adenomatous polyps in the colon as well as the rectum. A total proctocolectomy with ileal-anal pouch anastomosis was performed. The pathology of the specimen showed more than 100 colorectal adenomatous polyps, several of them showing carcinoma in situ and confirmed the diagnosis of FAP. 

**Figure 1:**
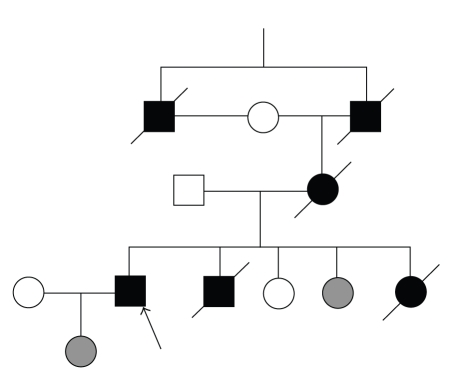
Partial pedigree of the family and mutation segregation. Square and circle symbols represent male and female, respectively. The genotypes of the exon ii polymorphism are shown under the symbols. Arrow: proband. Grey symbols: asymptotic carriers of the mutation.

The patient's mother previously died of colon cancer at the age of 39 years as well as his father and paternal uncle of his mother. This patient has one sister and one brother who died at age 19 and 27 years, respectively. In his family, he also has one sister and one daughter who have the mutation gene but are asymptomatic (Figure 1). 

The mutation screening showed heterozygous mutation in exon 15 at the codon 2016-2017 Del AT, p. Ser 672fsX5 at the extreme 5* end of the gene, the adenomatous polyposis coli (APC) gene. 

**Figure 2:**
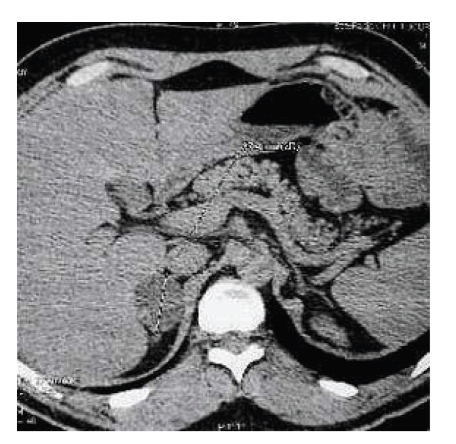
CT scan of the abdomen demonstrating a 2.5 × 4.0 cm mass in the right adrenal gland.

After being operated on, he began regular followup endoscopic examinations of intestinal and gastroduodenal polyps, which were insufficiently destroyed as they rose. The pathological evaluation revealed a tubulovillous adenoma with high-grade dysplasia. A routine abdominal computed tomography (CT) scan incidentally identified a 2.5 × 4.0 cm right adrenal (Figure 2). 

This patient was referred to our endocrinologist outpatients for exploration. 

Physical examination was unremarkable except for a tumefaction of the left mandible. His blood pressure was 130/75 mm Hg. His Weight was 92 Kg, his height 1,77 m, and his body mass index was 30,8 Kg/m².

The neurologic examination and the remainder of the systemic examination were normal. Signs of Cushing's syndrome were not noted. Chemistry profile was unremarkable (Table 1).

Endocrinological data showed alterations of the normal diurnal variation of cortisol (Table 2). Cortisolemia was not suppressed by 0,5 mg of dexamethasone given every 6 hour for 48 hours (plasma cortisol level was 14,4 *μ*g/dL after suppression). Computerized tomography adrenal scan identified bilateral masses, with a spontaneous density at 6 UH. These masses have homogeneous density and measured 3,5 cm in the right adrenal and 1 cm in the left adrenal. These criteria were in favor of benign masses. Therefore, this patient was found to have bilateral adrenocortical mass. The functional analyses showed a hormonal secretion pattern consistent with pre-Cushing's syndrome. Despite the character of secreting masses that indicate surgery, we opted for observation and recommended a 1-year interval CT. In three months, the masses did not changed in size or character, and given the risk of perioperative complications, observation was again recommended. The patient continues to fare well. 

**Figure 3:**
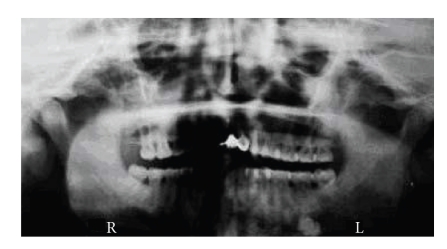
Panoramic radiograph shows an osteoma of the left angle of the mandible.

Our patient had another extra intestinal manifestations including:

- osteoma in the mandible without impacted or unerupted teeth objectified in the panoramic radiograph (Figure 3);
- a typical hypertrophy of the pigmented layer of the retina;
- the dermatological finding revealed histiocytofibroma in the right leg which was surgically removed.

## 3. Discussion

Gardner's syndrome, a variant of familial adenomatous polyposis, represents a multisystemic disease and disorder of growth [5].

The primary risk for patients with FAP and its variants is the development of colorectal cancer; however, there is also an increased incidence of other tumors, including adrenal masses. 

The polyp formation starts at puberty but diagnosis is usually made in the third decade, while the malignant transformation reaches 100% by the fourth decade of life [3]. 

Although not common, endocrine neoplasms such as parathyroid, pituitary, pancreatic islet cell, and adrenal neoplasms have all been described in patients with GS [2]. The first case of a FAP patient with an adrenal adenoma was published almost a century ago [4]. As a result of technological advances in imaging techniques such as CT and MRI during the last decades, new data have become available regarding the prevalence of adrenal masses in both the general population and patients with FAP. 

7% of patients with FAP or its variants have adrenal masses, compared with only 3% of the general public [4, 6]. Although the prevalence of adrenal masses in FAP patients are two to four times as high as in the general population, the clinical presentation and biological behavior do not seem to be different [4, 7].

Most adrenal lesions are not functional. Functional lesions typically secrete cortisol. Because most endocrine-associated tumors in patients with GS occur without symptoms, most cases are discovered incidentally or at autopsy. A review of the literature resulted in the identification of 17 cases of adrenal neoplasms (13 adenomas, 4 carcinomas) in patients with GS [8]. 

Only 4 patients, however, were symptomatic. They developed weight gain, hypertension, and headaches but did not have electrolyte abnormalities. Two of the patients had adrenal cortical carcinomas, and 2 had adrenal adenomas [8]. 

In all the 4 cases, the symptoms were consistent with cortisol hypersecretion or adrenal Cushing's syndrome [8].

While the overwhelming majority of these masses are benign and nonfunctional, there are reports of more aggressive and functional tumors in patients with FAP or its variants [9, 10].

Such rare cases are uncommon and highlight the relative risks of adrenal tumors versus other risks associated with treatment for FAP: one study of 132 FAP patients found that only one patient (0,9%) died of adrenal carcinoma, while 4,5% died from perioperative complications as a result of various abdominal operations [11].

Although the natural history is similar to lesions occurring sporadically, familial adenomatous polyposis-associated adrenal incidentaloma should warrant long term followup.

 In this rare condition, the development of a rigorous regimen will require evidence from worldwide patient cohorts. However, a tailored schema is suggested as a consistent basis for future modification [7]. Data on genetic analysis are limited, and only three mutations have been described (codons 1061, 1542, and 1981). The latter was associated with multiple and bilateral adenomas [4].

## 4. Conclusion

In conclusion, we presented a case of GS with an unusual clinical presentation of an adrenal tumor incidentally discovered. The hormonal finding confirmed the pre-Cushing's syndrome; the computed tomography showed bilateral adrenal masses and were in favor of the benign. Surgery was indicated for our patient, but we opted for observation, regularly taking into account the risk of perioperative complications. However, our patient is likely to develop complications from Cushing's syndrome.

Adrenal tumors were more common in FAP than in the general population, but require the same followup.

## Figures and Tables

**Table 1:** Chemistry profile of our patient.

Chemistry profile	
Creatinine = 83,2 *μ*mol/L	
Urea = 3,84 mmo/L	
Serum electrolytes: natremia = 135,9 mmo/L, kalemia = 4,77 mmo/L	
Hemoglobin = 14,2 g/100 mL	
White blood cell blood = 5910 plaquette = 160.000	
Cholesterol = 3,92 mmol/L triglyceride = 1,16 mmol/L	
Calcium = 2,4 mmol/L	
Phosphorus = 1,15 mmol/L	

**Table 2:** Endocrinological data of our patient.

Plasma cortisol at 8:00 AM = 12,7 *μ*g/dL (normal range: 6,2–19,4 *μ*g/dL)	
Plasma cortisol at 16:00 = 11,5 *μ*g/dL	
ACTH level = 12,2 pg/mL (normal range 10–50).	
Plasma aldosterone = 72, 7 pM/L (normal range: 274 PM/l) = 26 pg/mL	
Rennin was = 14,5 ng/L (normal range: 8,5 ng/L)	
Urinary metanephrines = 20.00 nmol/cr (normal range: 10–200)	
Urinary normetanephrine = 96.00 nmol/cr (normal range: 40–250)	
